# Combined Effects of Lactic Acid Bacteria and Protease on the Fermentation Quality and Microbial Community during 50 Kg Soybean Meal Fermentation Simulating Actual Production Scale

**DOI:** 10.3390/microorganisms12071339

**Published:** 2024-06-30

**Authors:** Huili Pang, Xinyu Zhang, Chen Chen, Hao Ma, Zhongfang Tan, Miao Zhang, Yaoke Duan, Guangyong Qin, Yanping Wang, Zhen Jiao, Yimin Cai

**Affiliations:** 1 School of Agricultural Sciences, Zhengzhou University, Zhengzhou 450052, China; pang@zzu.edu.cn (H.P.); aczhangxinyu@163.com (X.Z.); mahaoworks@foxmail.com (H.M.); tzhongfang@zzu.edu.cn (Z.T.); miaozhang@zzu.edu.cn (M.Z.); duyk@nwafu.edu.cn (Y.D.); qinguangyong@zzu.edu.cn (G.Q.); wyp@zzu.edu.cn (Y.W.); 2 School of Life Sciences, Zhengzhou University, Zhengzhou 450052, China; 13323825730@163.com

**Keywords:** soybean meal, production scale system, lactic acid bacteria, fermentation quality, microbial community

## Abstract

The improvement in the utilization rate and nutritional value of soybean meal (SBM) represents a significant challenge in the feed industry. This study conducted a 50 kg SBM fermentation based on the 300 g small-scale fermentation of SBM in early laboratory research, to explore the combined effects of lactic acid bacteria (LAB) and acid protease on fermentation quality, chemical composition, microbial population, and macromolecular protein degradation during fermentation and aerobic exposure of SBM in simulated actual production. The results demonstrated that the increase in crude protein content and reduction in crude fiber content were considerably more pronounced after fermentation for 30 days (d) and subsequent aerobic exposure, compared to 3 d. It is also noteworthy that the treated group exhibited a greater degree of macromolecular protein degradation relative to the control and 30 d of fermentation relative to 3 d. Furthermore, after 30 d of fermentation, adding LAB and protease significantly inhibited the growth of undesired microbes including coliform bacteria and aerobic bacteria. In the mixed group, the microbial diversity decreased significantly, and Firmicutes replaced Cyanobacteria for bacteria in both groups’ fermentation.

## 1. Introduction

Soybean meal (SBM), a by-product of soybean oil extraction, serves as one of the significant plant protein components in livestock and poultry feed due to its high protein content, balanced amino acids, and comprehensive nutritional elements, while also finding applications in producing pastries, health foods, cosmetics, and antibiotics [1]. According to different varieties, the SBM yield accounts for approximately 80% of soybeans; global SBM production approached 250 million tons in the year 2020/21, and among them, China is the main producer and consumer of SBM in the world [2]. Although the protein content of SBM is high, the large molecule protein is not easily absorbed by animals, and undigested proteins are fermented by intestinal bacterial spoilage, which produces several metabolites that are detrimental to health, such as ammonia, amines, phenolic compounds, and sulfide, etc. [3]. Additionally, the complex microflora of the SBM raw material complicates its storage, leading to a reduction in nutrient content and alterations in its components [4]. 

Current treatment methods for SBM include heat treatment, mechanical processing, chemical treatment, enzyme preparation, and microbial fermentation. Heat treatment is only suitable for antinutritional factors that are not stable to heat, and excessive heat treatment will destroy amino acids and vitamins. Heat treatment is only suitable for antinutritional factors that are not stable to heat, and excessive heat treatment will destroy amino acids and vitamins such as lysine, methionine, cysteine, tryptophan, and vitamins C, B1, A and E. thus, reducing the nutritional value of the feed [5]. The mechanical processing method is not widely used, but this also leads to increased feed costs. The chemical method includes residues that may be toxic to animals, meaning that the nutritional value of SBM is reduced, coupled with the high cost of chemical substances, resulting in this method not being applied frequently [6]. Although enzymes can effectively improve the nutritional value of SBM because they belong to the protein itself, the processing of high temperature can reduce their activity or directly inactivate them; in addition, adding too many will disturb the normal digestive function of the animal [7].

Fermentation of SBM by using microorganisms including *Aspergillus* (*A*.) *oryzae*, lactic acid bacteria (LAB), *Bacillus* spp., and yeast to degrade antinutritional factors, and the accumulation of beneficial metabolites can greatly improve the nutritional value of SBM, for this method can degrade macromolecular protein into protein peptides, and increase the protein level and digestibility, at the same time, it is nonpolluting, safe and reliable [8,9,10]. Hong et al. [11] used *A. oryzae* to ferment SBM, which broke down the antinutritional factors and increased the small molecule peptides. It has been shown that small molecule peptides have a variety of biological activities, such as immunostimulation, anti-hypertension, and anti- thrombosis, and can delay the increase in lactic acid in the blood and increase the storage of hepatic glycogen [12,13].

In microbial fermentation, LAB are one of the most commonly used and safest strains [14,15,16]. Protease-producing LAB and protease can degrade 7S and 11S globulins in SBM into small molecule proteins. LAB include *Lentilactobacillus* (*L.*) *plantarum* [17], *L. casei/paracasei* [18], *L. acidophilus* [19], *L. salivarius* [20], *Enterococcus* (*E.*) *faecium* [21] and *Pediococcus pentosaceus* [22], are widely used in SBM fermentation. *E. faecium* is a part of the normal flora in human and animal intestines, which can regulate the structure of animal intestinal flora, improve digestive enzyme activity, animal production performance, immunity and blood biochemical indices [23]. In addition to degrading antinutritional factors, LAB can also degrade oligosaccharides such as raffinose and fructose in SBM and produce lactic acid, increasing the levels of organic acids, reducing pH, and improving the flavor and palatability of feeds through the production of α-galactosidase [24]. Moreover, mixed fermentation with bacteria and protease has been shown to have a significant effect on the degradation of macromolecular protein, the shortening of the antigen degradation cycle, and the improvement in the nutritional quality and palatability of fermented soybean meal (FSBM) [25,26].

The process of SBM fermentation involves many microorganisms; once the fermentation is completed and feeding begins, it inevitably comes into contact with the air, disrupting the balance and causing a large number of aerobic microorganisms, such as yeast and mold, to actively reproduce, producing a series of harmful secondary metabolites [27]. In the current research on FSBM, studies have been mainly concentrated on small-scale fermentation in the laboratory [28]. For the actual production scale of soybean meal fermentation, such as the 50 kg scale, compared to the 300 g small-scale fermentation in the laboratory, the microorganisms involved during the fermentation process are different, and the pressure and storage problems faced after opening are also more severe. However, there is currently no relevant literature reporting on the specific situation. Therefore, based on small-scale fermentation, this study continues to carry out a 50 kg system of simulated actual production to explore the synergistic effect of LAB and protease on the actual production of FSBM to provide a practical and valuable reference for actual production.

## 2. Materials and Methods

### 2.1. Materials and Microorganisms

Soybean meal (SBM, 43.29% CP and 4.05% CF) and acid protease were purchased from China Grain and Oil ((Tangshan) Co., Ltd. (Tangshan, China) and Beijing Solarbio Science & Technology Co, Ltd. (Beijing, China)) respectively. *E. faecalis* was cultured at 30 °C in MRS (Man Rogosa Sharp) broth for overnight incubation.

### 2.2. Soybean Meal Fermentation Experiment 

According to the previous study [28], in order to keep the optimum fermentation humidity, the experiment was divided into control (50% SBM + 50% water) and treatment (50% SBM + 39% water + 10% ZZUPF95 (10^8^ CFU/mL) + 1% acid protease). Each group consisted of three replicates with an actual production weight of 50 kg, and all bags were fermented at room temperature (15–24 °C). Aerobic exposure was initiated after 3 and 30 days (d) of fermentation and continued for 1, 2, and 3 d, which were recorded as 3-1 d, 3-2 d, and 3-3 d, and 30-1 d, 30-2 d, and 30-3 d, respectively. The three-point method was used to sample after each opening to analyze the fermentation quality, chemical composition, and microbial community.

### 2.3. Fermentation Quality, Chemical Composition, and Microbiology Population Analysis

#### 2.3.1. Evaluation of Sensory

The sensory evaluation in this study was conducted using the procedure outlined by Meinlschmidt et al. [29]. A descriptive sensory analysis was conducted after unsealing the fermented soybean meal (FSBM) samples. The samples were divided into equal portions, and a panel of six evaluators assessed the color, odor, and tactile properties of both SBM (nonfermented soybean meal) and FSBM at various fermentation durations, including 3 and 30 d, as well as after 1, 2, and 3 d of aerobic exposure. The panel were trained by experts, evaluated the characteristics of the color, odor and structure of the FSBM by using observation with eyes, olfaction with nose, and twisting and rubbing with hands, respectively.

#### 2.3.2. Fermentation Quality

The pH of the FSBM filtrate was measured using a glass electrode pH meter (Mettler Toledo Co., Ltd., Greifensee, Switzerland). Lactic acid (LA) and acetic acid (AA) were quantified using HPLC (Waters 2695 HPLC system, Waters Technology Co., Ltd., Milford, CT, USA) with a Symmetry C18 column (4.6 × 250 mm, 5 µm). The mobile phase consisted of 25.4% vitriol at a flow rate of 0.6 mL/min with a column temperature of 55 °C. Elution absorbance was monitored at 214 nm [30,31].

#### 2.3.3. Chemical Composition

The dry matter (DM) content was determined by drying the samples in a forced-air oven at 65 °C for 48 h (h) until a constant weight was achieved. The AOAC standard method was used to determine the content of crude protein (CP) and crude fiber (CF) [32].

#### 2.3.4. Microbiology Population

Microbiological analysis was conducted using the method described by Pang et al. [33]. Each 10 g sample was diluted by combining with distilled water in a volume equivalent to 9 times its weight, and then, gradient dilution was performed. The 10^−3^, 10^−5^, and 10^−7^ diluted samples were then inoculated in triplicate onto various agar plates, respectively. Yeast and mold colonies were cultured on PDA agar and incubated at 30 °C, the difference is that the mold plate added tartaric acid, while the yeast plate did not. LAB, coliform bacteria, clostridia, and bacilli were incubated on MRS, EMB, CLO, and NA agar at 37 °C, with LAB and clostridia being cultured in anaerobic, and coliform bacteria and bacilli being cultured in aerobic. The entire cultivation time was 48 h [34,35].

### 2.4. SDS-PAGE Profile Determination

SDS-PAGE (sodium dodecyl sulfate–polyacrylamide gel electrophoresis) was used to assess protein degradation in FSBM [36]. Stacking and separating gels were prepared with acrylamide concentrations of 4% and 12%, respectively. The samples were heated at 100 °C for 10 min before electrophoresis. After electrophoresis, the gels were stained with Coomassie Brilliant Blue R250 to visualize and analyze protein degradation.

### 2.5. Microbial Community Measurement

#### 2.5.1. DNA Extraction

During fermentation, changes in microbiota, including bacterial and fungi communities and structures, were examined using advanced high-throughput sequencing. To obtain the microorganism suspension, 20 g of fermented SBM samples were mixed with 180 mL of sterile 0.85% NaCl solution. The mixture was agitated at 100 rpm for 2 h in an inspissator. Then, the suspension was filtered through a 0.22 mm sterile membrane and collected in a 2 mL microcentrifuge tube, and DNA extraction was performed using a DNA Kit provided by Shanghai Lian Shuo Biotechnology Co. Ltd. in Shanghai, China. The resulting DNA was eluted to a final volume of 80 µL. The DNA quantity, concentration, and quality were assessed using 1% agarose gel electrophoresis and by measuring the OD260 to OD280 nm ratio on a Thermo Scientific microplate reader in Wilmington, NC, USA. DNA samples that met the predetermined criteria were stored at −20 °C for further research.

#### 2.5.2. PCR Amplification

Following DNA extraction, the V3-V4 region of bacterial 16S rRNA was amplified using the following primers: 515F (5′-GTGCCAGCMGCCGCGGTAA-3′) and 806R (5′-GGACTACHVGGGTWTCTAAT-3′). For fungi DNA, ITS1F (5′-CTTGGTTCATTTAG AGGAAGTAA-3′) and ITS2-2043R (5′-GCTGCGTTCTTCATCGATGC-3′) were used to amplify the fungi ITS sequence. The amplification reaction consisted of a 25 µL mixture containing 25 ng of genomic DNA extract, 12.5 µL of PCR premix, 2.5 µL of primers, and 25 µL of water. The PCR conditions included an initial denaturation step at 98 °C for 30 s, followed by 35 cycles of denaturation at 98 °C for 10 s, annealing at 50 °C for 30 s (for bacteria) and 56 °C (for fungi), and extension at 72 °C for 45 s. An additional extension step at 72 °C for 5 min concluded the PCR process. Each DNA sample underwent three replicates, and the resulting PCR products were pooled together. The pooled PCR products were separated via 1% agarose gel electrophoresis and purified using a nucleic acid purification kit provided by Shanghai Lian Shuo Biotechnology Co. Ltd. in Shanghai, China. Subsequently, high-throughput sequencing was performed by Shanghai Applied Protein Technology Co., Ltd. (Shanghai, China).

The purified PCR product samples were sequenced using the IlluminaHiSeq2500 platform at Shanghai Zhongke New Life Biotechnology Co., Ltd. (Shanghai, China). Sequence analysis was performed using Uparse software (Uparse v7.0.1001, http://drive5.com/uparse/ accessed on 26 May 2024). Sequences with 97% similarity were assigned to the same operational taxonomic units (OTUs). The richness and diversity of the microbial indices were analyzed using QIIME (Version 1.7.0) with the Chao1 and Shannon index (http://www.mothur.org/wiki/Chao accessed on 26 May 2024). A heat map illustrating the correlation between microorganisms and fermentation quality was generated using R software (Version 2.15.3). Principal coordinate analysis and microbial community abundance figures were generated using the x-class website (http://www.biomicroclass.com accessed on 23 May 2024) for further analysis.

### 2.6. Statistical Analysis 

The experiments were carried out in specialized fermentation barrels, partitioned to refer to 6 periods, and the correlation between fermentation quality and microbial community was analyzed by Version 3.3.1 and 2.4.3. Each experiment was repeated three times to ensure that the results were reliable and consistent. The obtained data were analyzed using SPSS software, specifically Version 22. An analysis of variance (ANOVA) was conducted to assess the statistical significance of the observed differences. Duncan’s multiple range tests were employed to determine significant differences, with a threshold of *p* < 0.05.

## 3. Results

### 3.1. Sensory Evaluation

For the sensory evaluation of 50 kg FSBM, after both 3 and 30 d of fermentation, compared to the control group, the ZZUPF 95- and protease-treated group (group H) maintained a similar color, fluffy texture but a stronger sour aroma than the original SBM. After fermentation 3 d, the control group began to show localized agglomeration when aerobic exposure for 1 d, and produced a strong malodorous odor and large agglomerated coagulation at 3 d aerobic; but after 30 d fermentation, agglomeration and localized mold did not appear until 30 d-3 d. While in group H, only slight molds at 3 d-3 d were observed, and the aerobic exposure after 30 d of fermentation always maintained better looks and smells.

### 3.2. The Fermentation Quality

**Figure 1:**
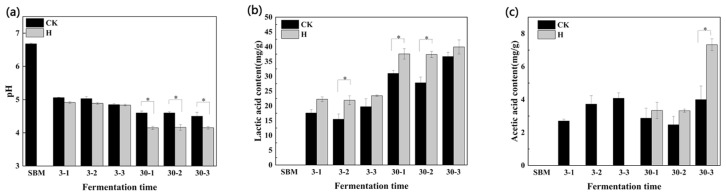
Effects of pH, lactic acid, and acetic acid among control and treatment groups aerobic exposure for 1–3 days after 3 and 30 days fermentation. * indicates significant differences (*p* < 0.05). (**a**), the pH value; (**b**,**c**), the lactic acid and acetic acid content, respectively. CK, the control group (50% SBM + 50% water). H, the treatment group (50% SBM + 39% water + 10% ZZUPF95 (10^8^ CFU/mL) + 1% acid protease). SBM, soybean meal. d, days. Respectively, 3-1, 3-2, and 3-3 and 30-1, 30-2, and 30-3, represent aerobic exposure 1 d, 2 d, and 3 d after fermentation at 3 d and 30 d.

The fermentation treatment of the SBM resulted in a reduction in pH value compared to the original sample in both the control and treated groups (Figure 1). There was no significant difference in the pH value of the control group within 30 d of fermentation (*p* > 0.05). However, the pH values of the H group were significantly higher than the initial pH after 3 d of fermentation (*p* < 0.05), but these subsequently decreased to below 4.20. After 3 d of fermentation, there was no significant difference in pH between the control and treatment groups (*p* > 0.05). However, the pH of the H group remained lower than that of the control group, and the pH of the H group decreased to below 5.00 during all fermentation times.

As for the 30 d fermentation period, the pH value of the H group decreased the most and was significantly lower than that of the control group (*p* < 0.05). During the 30-2 d exposure period, the pH value was the lowest that it reached, 4.16, in the entire fermentation stage.

Regarding lactic acid changes, Figure 1b indicates that lactic acid levels increased after 30 d of fermentation compared to 3 d, with the H group displaying significantly higher levels than the control group during 30-1 and 30-2 period (*p* < 0.05). At 30-3 d, the lactic acid in the H group peaked at 39.30, the highest observed throughout the experiment. The group H consistently exhibited higher lactic acid content than the control, with significant differences observed at 3-2 d, 30-1 d, and 30-2 d (*p* < 0.05).

Figure 1c shows the changes in acetic acid content before and after fermentation and during aerobic exposure. Acetic acid was undetectable in the H group, and there was no statistically significant change in the control group on d 3 and 30 of fermentation (*p* > 0.05). Compared to the control, the H group had significantly higher acetic acid content only during the 30-3 day exposure (*p* < 0.05).

### 3.3. Chemical Composition Analysis

**Figure 2:**
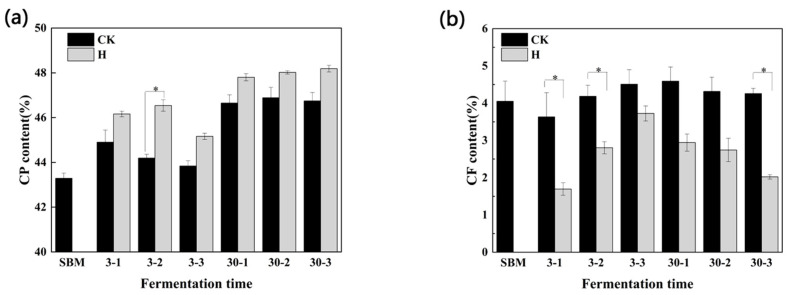
Effects of crude protein and crude fiber among control and treatment groups aerobic exposure for 1–3 days after 3 and 30 days fermentation. * indicates significant differences (*p* < 0.05). CP, crude protein. CF, crude fiber. SBM, soybean meal. (**a**), the crude protein; (**b**), the crude fiber. Respectively, 3-1, 3-2, and 3-3 and 30-1, 30-2, and 30-3, represent aerobic exposure 1 d, 2 d, and 3 d after fermentation at 3 d and 30 d. CK, the control group (50% SBM + 50% water); H, the treatment group (50% SBM + 39% water + 10% ZZUPF95 (10^8^ CFU/mL) + 1% acid protease).

As depicted in the Figure 2a, the CP content of both experimental groups following fermentation and aerobic exposure exhibited a statistically significant increase compared to the initial SBM composition (*p* < 0.05). Different fermentation duration had a significant effect on the CP content in SBM. The CP contents in both control and H groups were significantly higher at 30 d of fermentation compared to 3 d (*p* < 0.05). Additionally, the CP levels in the group H were consistently higher than that in the control, and with a significant increase observed at the second opening after 3 d of fermentation (*p* < 0.05).

Regarding the changes in CF during fermentation and subsequent aerobic exposure, Figure 2b demonstrates that the content of CF in the H group was lower than that in the control group throughout the 30 d fermentation process. Notably, when aerobic exposure for 1 d and 2 d after 3 d fermentation, and 3 d after fermentation for 30 d, CF in the group H were significantly lower than that in control (*p* < 0.05).

### 3.4. Microbial Population of FSBM

Throughout 30 d of fermentation and the subsequent aerobic exposure period (Table 1), the number of LAB in all the fermentation groups was significantly higher than the original SBM (*p* < 0.05), and the LAB in group H were higher than those in the control group throughout all the periods, where a concentration of 10^8^ CFU/mL was reached after 3 d of fermentation, significantly exceeding that of the control (*p* < 0.05). Moreover, LAB in all groups after 3 d of fermentation were higher than those in the corresponding groups at 30 d regardless of the control or treatment groups [37].

As for counts of unwanted microorganisms including coliform and aerobic bacteria, they were all lower in group H than that in the control group throughout the experiment. Coliform bacteria were significantly reduced in group H throughout the experiment, but aerobic bacteria were only significantly reduced in aerobic exposure 1 and 2 d after 30 d of fermentation.

### 3.5. SDS-PAGE Profile 

**Figure 3:**
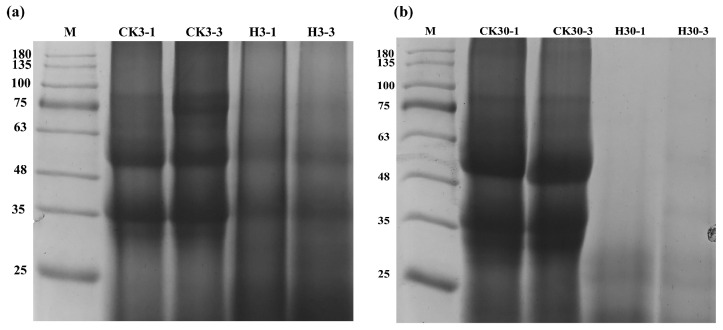
Protein degradation among control and treatment groups aerobic exposure for 1–3 days after 3 and 30 days fermentation. (**a**,**b**), protein degradation among control and treatment groups aerobic exposure for 1 and 3 days after 3 (**a**) and 30 days (**b**) fermentation, respectively. CK3-1, CK3-3, CK30-1, and CK30-3 and H3-1, H3-3, H30-1, and H30-3: control check and treated groups of aerobic exposure 1 d and 3 d after fermentation at 3 d and 30 d, respectively. CK, the control group (50% SBM + 50% water). H, the treatment group (50% SBM + 39% water + 10% ZZUPF95 (10^8^ CFU/mL) + 1% acid protease).

During the aerobic exposure process after 3 d of fermentation (Figure 3), the protein bands in the control group were concentrated at 35–75 kDa, while the bands in group H were mainly below 48 kDa, and the color of the bands was significantly lighter than that of the corresponding control. After 30 d of fermentation, the protein bands in the control group remained concentrated at 25–48 kDa during aerobic exposure, mainly at 35 and 48 kDa. There was no significant difference between aerobic exposure for 1 d and 3 d. In the H group, bands were found at 25 kDa only after 1 d of aerobic exposure, and the imprint was not obvious.

### 3.6. Microbial Community Dynamics

#### 3.6.1. Alpha Index

**Figure 4:**
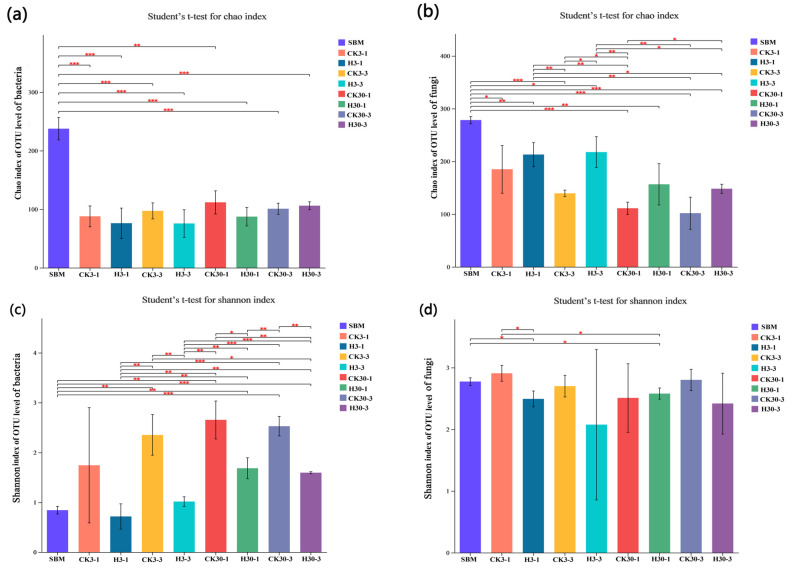
Alpha index of microbes among control and treatment groups aerobic exposure for 1 and 3 days after 3 and 30 days fermentation. (**a**,**b**) Chao index of bacteria and fungi; (**c**,**d**) Shannon index of bacteria and fungi. * indicates significant differences (*p* < 0.05), ** indicates *p* < 0.01, *** indicates *p* < 0.001. SBM, soybean meal, CK3-1, CK3-3, CK30-1, and CK30-3 and H3-1, H3-3, H30-1, and H30-3: control checks group and treated group of aerobic exposure at 1 d and 3 d after fermentation at 3 d and 30 d, respectively. CK, the control group (50% SBM + 50% water). H, the treatment group (50% SBM + 39% water + 10% ZZUPF95 (10^8^ CFU/mL) + 1% acid protease).

The abundance of both bacteria and fungi changed significantly before and after SBM fermentation, with a significant reduction in abundance being observed (*p* < 0.001) in Figure 4. The bacterial abundance in group H did not show significant changes during each period, but it was consistently lower than that of the control group, except for the 3 d of aerobic exposure after 30 d of fermentation. In the fungi community, the abundance of each group was reduced significantly compared to the original SBM, but the abundance of the fungi community increased in group H compared to the control group (Figure 4a,b).

From the Shannon index of bacterial (Figure 4c) and fungi (Figure 4d) community diversity for each treatment group during fermentation and aerobic exposure, throughout each stage, group H consistently exhibited lower bacterial diversity compared to the control group. The results obtained for bacterial community diversity further support this trend. The fungi diversity in group H almost showed the same results as bacteria, except for the 30-1 d of aerobic exposure.

#### 3.6.2. Principal Coordinate Analysis

**Figure 5:**
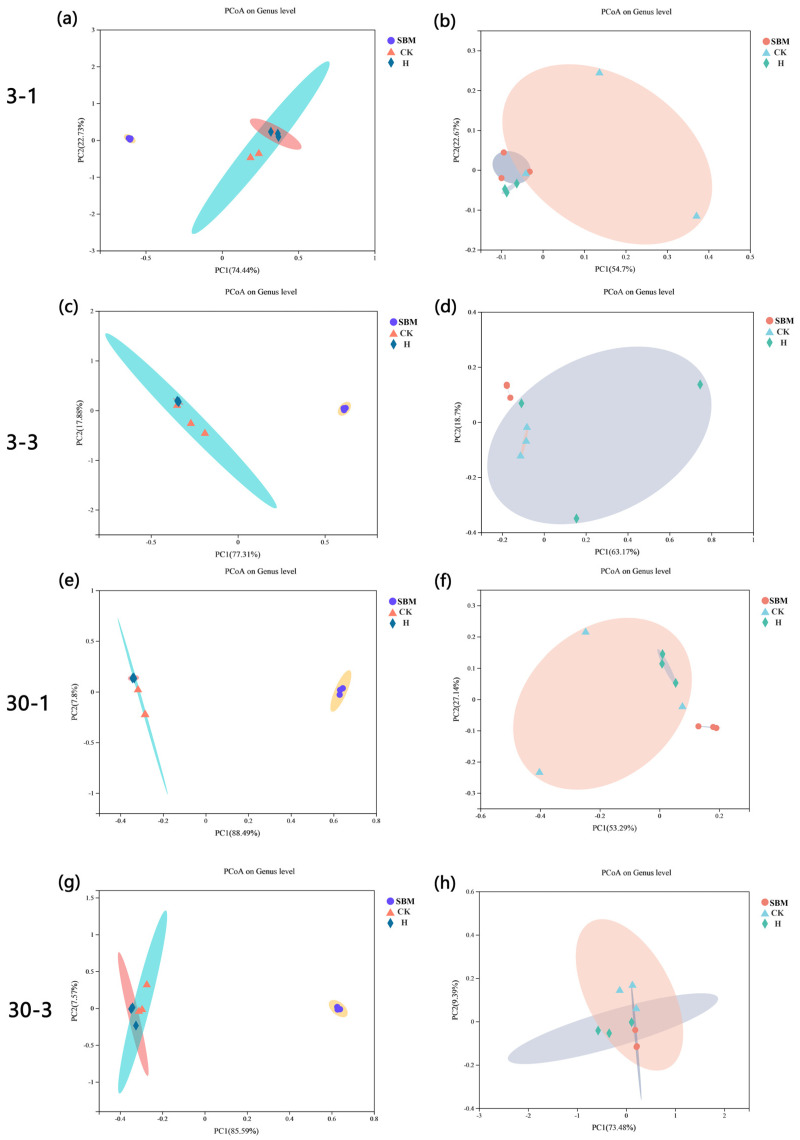
Principal coordinate analysis on genus level of bacterial (**a**–**d**) and fungal (**e**–**h**) communities among control and treatment groups aerobic exposure for 1 and 3 days after 3 and 30 days fermentation. SBM, soybean meal; CK, the control group (50% SBM + 50% water). H, the treatment group (50% SBM + 39% water + 10% ZZUPF95 (10^8^ CFU/mL) + 1% acid protease). Respectively, 3-1, 30-1, 3-3, and 30-3: aerobic exposure 1 d and 3 d after fermentation at 3 d and 30 d.

In the bacterial community analysis, the control and H groups were significantly separated from the FSBM group compared with the original SBM group, and the bacterial community structure of the control group and the H group was similar at 30-1 d of aerobic exposure (Figure 5c), while differences occurred in the other three periods (Figure 5a,b,d).

In the analysis of the fungi community, notable distinctions were found between the H, CK, and SBM groups. However, the most prominent differences emerged after 1 and 2 d of aerobic fermentation following 3 d of fermentation (Figure 5e,f).

#### 3.6.3. Abundance of Microbial Community

**Figure 6:**
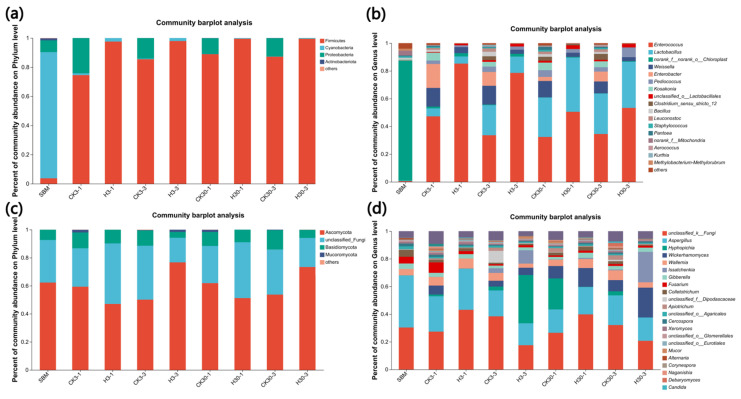
Microbial community among control and treatment groups aerobic exposure for 1 and 3 days after 3 and 30 days fermentation on phylum and genus levels. (**a**,**c**), the bacterial and fungi communities during fermentation and aerobic exposure on the phylum level; (**b**,**d**), the bacterial and fungi communities during fermentation and aerobic exposure on the genus level. SBM, soybean meal. CK3-1, CK3-3, CK30-1, and CK30-3 and H3-1, H3-3, H30-1, and H30-3: control checks group and treated group of aerobic exposure at 1 d and 3 d after fermentation at 3 d and 30 d, respectively. CK, the control group (50% SBM + 50% water). H, the treatment group (50% SBM + 39% water + 10% ZZUPF95 (10^8^ CFU/mL) + 1% acid protease).

At the phylum level (Figure 6a), SBM was dominated by Cyanobacteria (86.87%). Following fermentation treatment, Firmicutes became the dominant bacteria in both groups, with a higher proportion in group H compared to the control group. After 30 d of fermentation, the proportions of Firmicutes in 30-1 and 30-2 reached 99.48% and 99.46%, respectively.

At the genus level (Figure 6b), *Enterococcus* was the dominant flora in all FSBM groups, except for the original SBM, and the proportion in group H was higher than that in the control group, which was still the highest proportion (85.24%) observed after 3-1 d exposure. Additionally, there was a high proportion of *Lactobacillus* in all groups, especially after 30 d of fermentation, with proportions of 39.09% and 33.26% in the CK and H groups, respectively.

Regarding fungal community abundance, Ascomycota was the dominant phylum in each group at the phylum level (Figure 6c), with the proportion of this phylum reaching the highest level (76.67%) when exposed for 3 d after 3 d of fermentation.

For the genus level (Figure 6d), *Candida* was an important genus in all groups during the aerobic exposure phase after 30 d fermentation, while it was higher in group H than in the control. The dominant genus was *Hyphopichia* for the 3-3 H group, *Issatchenkia* occupied an important position in 3 d exposure both after 3 and 30 d fermentation, and the highest proportion (37.64%) of *Aspergillus* was observed in the SBM.

### 3.7. Correlation Analysis between Fermentation Quality and Microbial Community

**Figure 7:**
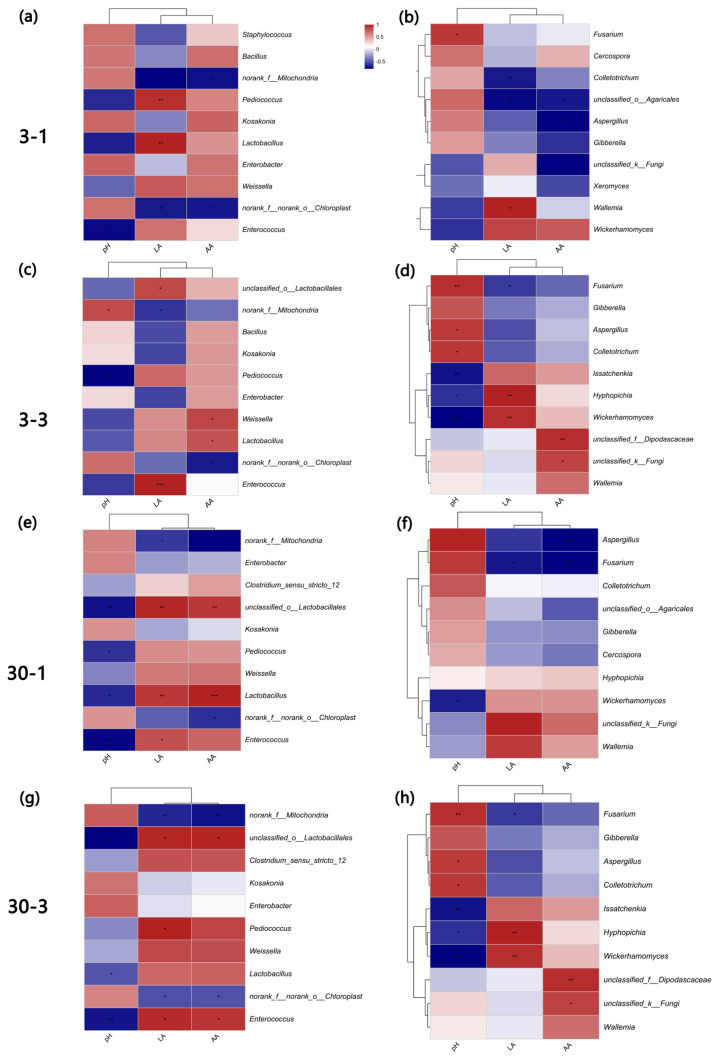
Spearman correlation heatmap of abundance between the top 10 most enriched bacterial (**a**–**d**) and fungal (**e**–**h**) communities during control and treatment groups aerobic exposure for 1 and 3 days after 3 and 30 days fermentation. * indicates significant differences (*p* < 0.05), ** indicates *p* < 0.01, *** indicates *p* < 0.001. LA, lactic acid; AA, acetic acid. SBM, soybean meal. CK3-1, CK3-3, CK30-1, and CK30-3 and H3-1, H3-3, H30-1, and H30-3: control checks group and treated group of aerobic exposure at 1 d and 3 d after fermentation at 3 d and 30 d, respectively. CK, the control group (50% SBM + 50% water). H, the treatment group (50% SBM + 39% water + 10% ZZUPF95 (10^8^ CFU/mL) + 1% acid protease).

It can be seen that no significant correlation existed between fermentation quality and bacteria when exposed for 1 d after 3 d of fermentation (Figure 7a); however, after 3 d exposure, *Lactobacillus* exhibited an extreme correlation with pH (*p* < 0.01), and *Pediococcus* displayed an incredibly significant (*p* < 0.001) negative correlation with pH (Figure 7b). Additionally, both *Lactobacillus* and *Enterobacter* were significantly negatively correlated with pH (*p* < 0.01) after being exposed twice after 30 d of fermentation (Figure 7c,d). For fungi, the most significant negative correlation was observed between microorganisms with pH (*p* < 0.01), including *Wickerhamomyces* and *Mucor*.

Concerning organic acids, the lactic acid content showed a highly significant positive correlation with *Enterococcus* when exposed for 3 d after 3 d of fermentation (*p* < 0.001), and it showed an incredibly significant positive correlation with *Lactobacillus* for 1 d of exposure after 30 d of fermentation (*p* < 0.01). Moreover, *Lactobacillus* also displayed an extremely significant positive correlation with acetic acid content (*p* < 0.001) (Figure 7e,f). In the fungal correlation analyses, a significant positive correlation can be observed between the lactic acid content and *Hyphopichia*, *Wickerhamomyces*, and *Mucor* when exposed for 3 d after 3 d of fermentation, and a significant positive correlation was shown with *Issatchenkia* for 1 d after 30 d of fermentation (*p* < 0.01). The acetic acid content was significantly positively correlated with *Rhizopus* and *Mucor* in 1 d of exposure after 3 d of fermentation, and *Candida* was significantly positively correlated in 3 d of exposure after 30 d of fermentation, respectively (*p* < 0.01) (Figure 7g,h).

## 4. Discussion

In recent years, SBM has garnered considerable attention in the feed fermentation industry. Researchers have extensively utilized probiotics, such as LAB including *E. faecalis*, and *Bacillus* spp. that are known for their proteolytic capabilities and robust stress resistance, to enhance the solubility and intestinal digestibility of SBM during fermentation processes [38,39,40,41]. Despite numerous small-scale laboratory studies on SBM fermentation, there is a scarcity of research emulating large-scale production environments [42,43,44].

Findings of this research revealed that incorporating *E. faecalis* and protease into the SBM fermentation process significantly reduced the pH level, and this reduction became more pronounced over a 30 d period. Similar to these observations, Chen et al. [45] conducted SBM fermentation in 10 L jars and found fermented with *L. plantarum* FJAT-13737 exhibited the lowest pH value of 5.75 after 8 d. A lower pH environment inhibits the proliferation of pathogenic bacteria such as *Escherichia coli*, *Staphylococcus* spp., and *Clostridium* spp., which prefer neutral to alkaline conditions [46,47]. Concurrently, lactic acid levels increased, reaching peak concentrations at the end of experiment, which directly correlated with the observed pH reductions and enhanced antibacterial capabilities across a broad spectrum. In addition, this pattern was consistent with smaller-scale experiments (300 g) that *E. faecalis* and protease treatment groups showed notable increases in lactic acid, irrespective of the fermentation conditions—whether anaerobic or aerobic [28]. This suggests a qualitative enhancement in the fermentation process, underscored by an increased lactic acid content throughout the duration of their experiment, for substantial production of lactic acid not only fosters the growth of beneficial fermentation strains, but creates an acidic milieu in the animal’s gut, thereby limiting the growth and propagation of most pathogenic bacteria and molds. This contributes significantly to enhancing the animal’s disease resistance capabilities [48,49].

The fermentation process significantly influenced the content of CP and CF in SBM. Notably, CP content increased while CF content decreased after fermentation and aerobic exposure in the treated group in the present study. The increase in CP can be attributed to microbial respiration during fermentation, which consumes organic matter and releases carbon dioxide and water [46]; such a reduction in total mass leads to a “protein concentration effect”. It is worth mentioning that this not only improves the protein level of FSBM protein substrate, but plant proteins of SBM are metabolized by microorganisms into mycoproteins, which also changes the quality of the proteins in SBM in the fermentation process [47]. Furthermore, some of the increased protein is the result of the mycoprotein contained in the microorganisms’ bodies and the degradation of the CF components including cellulose and hemicellulose during the fermentation process by the microorganisms, which is the most meaningful part of the CP content increase in fermentation product. This accounts for the increase in CP and decrease in CF, and the phenomenon has also been observed in small-scale SBM fermentations reported by Wang et al. [48] and Ma et al. [28].

The process of fermentation is a dynamic reaction of enzymes and microorganisms such as LAB, bacilli and yeast, and the addition of LAB can accelerate the accumulation of lactic acid, lower the pH and inhibit the growth and reproduction of undesirable microorganisms [49]. In the present study, the number of various microorganisms decreased in both groups, and the growth of coliform and bacilli were inhibited much more obviously at 30 d of fermentation relative to 3 d in ZZUPF95 mixed protease-treated group. These are also the results of the low pH and high lactic acid content described earlier. As for LAB, which constitute the most important microbial population in fermented feeds, it was higher after 3 d of fermentation in all groups than in the corresponding groups at 30 d. This could be attributed to the fact that when the environmental pH drops to extremely low levels, LAB struggle to maintain intracellular pH stability, resulting in the intracellular pH begins to decline, eventually equalizing with the external pH; this equilibrium disrupts LAB’s physiological processes, leading to a cessation of growth and cell death ultimately [50]. In other words, extremely low pH inhibits even the growth of the LAB themselves.

After microbial fermentation treatment of SBM, the macromolecular proteins are decomposed into small peptides (peptides containing 2 or 3 amino acid residues). Small peptides can be completely absorbed and enter the circulatory system to be used by the tissues, improve protein utilization, reduce the chances of nutritional diarrhea, promote animal growth and reduce morbidity, thus improving the nutritional value and conversion rate of the feed [51]. The present study found, in a 50 Kg fermentation system, the effect of the ZZUPF95 + protease group on the degradation of large protein molecules was even more significant and pronounced during the period of fermentation and aerobic exposure compared to the control groups. Notably, as occurs with other indicators, as fermentation time prolongs, the protein degradation effect becomes more significant, which exhibited a greater effect on protein degradation in group H at 30 d compared to 3 d of fermentation. This is also consistent with the results showed by Hong et al. [11] that fermentation increased the content of small peptides <10 KDa in SBM, and 22.2% of the peptides in unfermented SBM were large peptides, while FSBM did not contain large peptides >60 KDa; and Shi et al. [52], who also found a significant increase in the number of small peptides <25 KDa in SBM by fermentation with *Bacillus subtilis*.

Alpha diversity includes the Shannon index and Chao1 index, etc., which reflect the species diversity and microbial abundance of individual samples. The Shannon index is commonly used to measure species diversity, while Chao1 is usually used to measure species abundance [53]. Group H consistently exhibited lower bacterial and fungi diversities compared to the control group, might be attributed to the relatively low pH in the FSBM, which combined with the acidifying activity, promotes the reduction in bacterial and fungal diversities and ultimately improves feed quality [54]. In the same way, after SBM fermentation, the abundance of both bacteria and fungi significantly reduced, indicating that LAB rapidly proliferated into the dominant species while other species were inhibited in a closed environment throughout the fermentation process [55]. The bacterial and fungal abundances in the treated group were always lower, which might be attributed to the action of lactobacilli and protease that resulted in relatively low pH. These results concur with reports that inhibition growth and reproduction of undesirable microbes in fermented feeds would reduce the abundance of microbial composition and thus improve the quality of fermented feeds [56].

Changes in key microorganisms during fermentation and aerobic exposure are closely related to feed quality [57]. Firmicutes became the dominant bacteria in both groups after fermentation, and the proportion in group H was higher compared to the control. The Firmicutes phylum, which includes many probiotic members like *Enterococcus* and *Lactobacillus*, plays a significant role in influencing metabolism and maintaining homeostasis of the internal environment through its specific flora structure, activity and metabolites [9]. Firmicutes are also known to degrade cellulose in anaerobic environments, which likely contributes to the observed CF changes in FSBM [58]. This finding aligns with results from both small-scale FSBM studies and larger silage studies involving whole crop corn, wheat, and wheat straw, and it was further verified that anaerobic conditions may favor the growth of Firmicutes as a dominant flora [59,60,61,62,63,64]. Notably, even after 1 to 2 d of aerobic exposure following 30 d of fermentation, the Firmicutes in the ZZUPF95 + protease-treated group reached peak levels, suggesting that this exposure did not significantly compromise FSBM quality. On the other hand, the dynamics of Gram-negative bacteria Cyanobacteria to Gram-positive Firmicutes during fermentation and aerobic exposure process, suggests that fermentation inhibits the proliferation of pathogenic bacteria that are widely present in SBM [65,66].

Ascomycota is one of the species-rich phyla of the fungi with about 11,000 species. Fermentation also promoted the dominance of the Ascomycota phylum in fungi within both control and treated groups. Genera such as *Tuber* spp. within this phylum are prolific carbohydrate producers, contributing raw materials crucial for ongoing fermentation processes. *Ramichloridium* spp., known for its cellulose-hydrolyzing ability, further supports the substrate and energy supply for fermentation [67,68]. Moreover, the addition of ZZUPF95 and protease, coupled with prolonged fermentation, significantly reduced the relative abundance of potentially harmful *Aspergillus* species, such as *Aspergillus flavus*, which are known for producing aflatoxins [69]. In addition, *Aspergillus* can be isolated from a wide range of materials worldwide, and highly growth-adapted with its optimal growth pH being close to that achieved in feed, with the prevalence in feed ranging from 8 to 75% [70,71]. This reduction enhances the safety and quality of the fermented product. *Hyphopichia* is present in traditional fermentations with aroma-producing ability and a potential role in the formation of flavor in fermented products [72]. *Issatchenkia* can synthesize pectinases and lipases [73], which are associated with the production of higher alcohols and volatile acids in fermentation. It has been claimed that Candida is a parthenogenetic anaerobic fungi that can survive under both aerobic and anaerobic conditions and that the acidic environment during fermentation allows the yeast cells to autolyze released vitamins and other nutrients that favor the growth of LAB [74]. There are also many studies showing that mixed fermentation of *Lactobacillus* and *Candida* helps to improve the organoleptic characteristics of feed color, aroma, and flavor [75]. This might contribute to the color and odor of FSBM together with *Hyphopichia.*

The present study exhibited pH which shows a high and incredibly significant negative correlation with organic acid, while organic acids, including lactic acid and acetic acid, are always positively correlated with LAB, including lactobacilli and Pediococci, after 3 d and 30 d of fermentation followed by aerobic exposure, respectively. LAB dominate fermentation and convert hexacarbon sugars containing six carbon atoms into lactic acid containing two parts of three carbon atoms, can accumulate lactic acid, lower the pH of the environment, inhibit the proliferation of harmful bacteria, and promote feed fermentation [66]. As for fungi positively correlated with organic acid, they were also confirmed by their individual characteristics. *Pichia* spp. has a good lactic acid tolerance, *Wickerhamomyces* spp. increases acid content, and *Mucor* spp. primarily converts proteins to amino acids; moreover, *Rhizopus* spp. has some acid-producing capacity, *Candida rugosa* can convert sugar to ethanol and break down pectin and the lipase it produces is widely used in the production of fatty acids [76,77,78]. Their specific role in fermented feed still needs further detailed verification.

## 5. Conclusions

In a 50 kg soybean meal fermentation system, compared to the control, across fermentation and aerobic exposure, the group treated with *Enterococcus faecalis* ZZUPF95 mixed acid protease exhibited significant decreases in pH value and CF content, while showing a relative increase in lactic acid and crude protein contents. The protein profile of the treated group degraded significantly compared to the control, especially after 30 d of fermentation. Furthermore, the population of lactic acid bacteria increased, while unwanted bacteria including coliform bacteria and aerobic bacteria were inhibited. A significant reduction in microbial diversity and changes in the abundance of bacteria and fungi occurred, with a significant increase in the abundance of *Lactobacillus* and *Enterococcus* also being discovered. The results of this study provide further reference for understanding changes in the fermentation process of soybean meal and exploring efficient additives in practical production.

## Figures and Tables

**Table 1:** Dynamic changes in microbial population through fermentation and aerobic exposure (lg cfu/g FM).

Item	Treatment	Days of Ensiling (d)						SEM	*p* Value
		3-1	3-2	3-3	30-1	30-2	30-3		
LAB	CK	9.88 ^Ba^	9.79 ^Ba^	9.33 ^Bab^	8.59 ^Abc^	8.43 ^Bbc^	7.86 ^Bc^	0.24	<0.001
	H	11.31 ^Aa^	10.92 ^Aa^	10.80 ^Aac^	9.41 ^Abc^	9.72 ^Ac^	9.21 ^Abc^		
Yeast	CK	4.06 ^Aa^	4.97 ^Aa^	5.51 ^Aab^	ND ^Aab^	ND ^Bab^	ND ^Bb^	0.90	<0.001
	H	ND ^Bb^	0.12 ^Aa^	0.36 ^Aa^	0.57 ^Aa^	0.15 ^Aa^	0.07 ^Aa^		
Bacilli	CK	0.10 ^Ac^	0.06 ^Ac^	0.03 ^Ac^	0.06 ^Aa^	0.14 ^Ab^	0.27 ^Aa^	0.18	<0.001
	H	0.06 ^Ab^	0.14 ^Ab^	0.13 ^Ab^	0.46 ^Aa^	0.07 ^Aa^	0.20 ^Ba^		
Coliform bacteria	CK	9.86 ^Aa^	9.89 ^Aa^	9.97 ^Aa^	7.74 ^Ab^	7.35 ^Ab^	7.26 ^Ab^	0.25	<0.001
	H	7.44 ^Ba^	5.57 ^Bc^	7.00 ^Bab^	5.92 ^Bbc^	5.46 ^Bc^	5.34 ^Bc^		
Aerobic bacteria	CK	9.93 ^Aa^	9.99 ^Aa^	9.62 ^Aa^	9.46 ^Aa^	9.00 ^Aa^	5.77 ^Ab^	0.28	<0.001
	H	9.72 ^Aa^	9.84 ^Aa^	9.44 ^Aa^	6.73 ^Bbc^	7.23 ^Bb^	5.57 ^Ac^		

^A,B^, differences between CK and H at the same time. ^a–c^, differences in different periods of the same group. LAB, lactic acid bacteria. Respectively, 3-1, 3-2, and 3-3 and 30-1, 30-2, and 30-3, represent aerobic exposure 1 d, 2 d, and 3 d after fermentation at 3 d and 30 d. CK, the control group (50% SBM + 50% water). H, the treatment group (50% SBM + 39% water + 10% ZZUPF95 (10^8^ CFU/mL) + 1% acid protease).

## Data Availability

Data are contained within the article.
